# Understanding Typology of Preexposure Prophylaxis (PrEP) Persistence Trajectories Among Male PrEP Users in the United States

**DOI:** 10.1093/ofid/ofae584

**Published:** 2024-10-11

**Authors:** Yi-No Chen, Junlan Zhou, Heather S Kirkham, Edward A Witt, Samuel M Jenness, Kristin M Wall, Rishi Kamaleswaran, Ashley I Naimi, Aaron J Siegler

**Affiliations:** Department of Epidemiology, Emory University, Atlanta, Georgia, USA; Health Analytics, Research, and Reporting Department, Walgreen Co., Deerfield, Illinois, USA; Health Analytics, Research, and Reporting Department, Walgreen Co., Deerfield, Illinois, USA; Health Analytics, Research, and Reporting Department, Walgreen Co., Deerfield, Illinois, USA; Department of Epidemiology, Emory University, Atlanta, Georgia, USA; Department of Epidemiology, Emory University, Atlanta, Georgia, USA; Department of Biomedical Informatics, Emory University, Atlanta, Georgia, USA; Department of Epidemiology, Emory University, Atlanta, Georgia, USA; Department of Epidemiology, Emory University, Atlanta, Georgia, USA

**Keywords:** Group-based trajectory model, HIV prevention, MSM, PrEP persistence, Medication adherence

## Abstract

**Introduction:**

Understanding longitudinal patterns of preexposure prophylaxis (PrEP) use among men who have sex with men could offer insights for developing efficient and timely interventions to promote PrEP persistence.

**Setting:**

We extracted 2 years of pharmacy fill records for 4000 males who initiated PrEP in 2017 at a national chain pharmacy in the United States.

**Methods:**

Group-based trajectory models were used to develop PrEP trajectory clusters, with periods of use defined based on optimal PrEP seroprotection probabilities (ie, PrEP use frequency ≥4 doses/week). Multinomial logistic regressions were used to evaluate the associations between sociodemographic covariates and identified trajectory group membership.

**Results:**

We identified 4 distinct groups of PrEP persistence trajectories: (1) persistent use of PrEP throughout the period (persistent user), (2) brief use followed by sustained cessation of PrEP use (brief user), (3) PrEP use up to the mid-term followed by sustained cessation of PrEP use (mid-term user), and (4) PrEP use, followed by cessation and subsequent reinitiation (PrEP reinitiator). Persistent users and brief users accounted for 40.1% and 22.9% of the population, respectively, whereas mid-term users and reinitiators accounted for 18.9% and 18.2%, respectively. Older age at PrEP initiation, commercial insurance as the primary payer of PrEP, and use of specialty pharmacy were found to be associated with persistent PrEP use over the other patterns of nonpersistence.

**Conclusions:**

Subgroups of PrEP users could benefit from PrEP persistence interventions that target specific timings of likely PrEP cessation or considerations of reinitiation.

## Teaser

Analysis of nationwide PrEP fill data revealed 4 use trajectories. Heterogenous timings of PrEP cessations and reinitiation suggest tailored interventions for persistence. Association of persistence with specialty pharmacy use and insurance underscores the importance of financial and medication adherence support.

Men who have sex with men (MSM) are the population most affected by HIV in the United States, accounting for approximately 69% of diagnoses [1] despite comprising ∼2% of the US population. Therefore, it is crucial to improve the effectiveness of HIV prevention strategies targeting MSM to reduce new infections. Since 2012, daily oral preexposure prophylaxis (PrEP) has been recommended for MSM at risk of HIV acquisition [2] because of its high efficacy in randomized controlled trials [3, 4]. However, the effectiveness depends on persistence over time; PrEP use of at least 4 doses per week provides high levels of protection [5].

Limited persistence in PrEP care poses a substantial challenge to its success as an HIV preventive measure among MSM. Data from PrEP clinics indicated that only 57% of MSM remained in PrEP care 6 months after initiation [6]. A later multisite review of clinical records suggested that only 30% of MSM persisted for 12 months [7]. Despite a critical need to develop effective strategies to improve PrEP persistence, few observational studies have analyzed persistence over extensive follow-up periods. There may be benefits to understanding PrEP persistence as dynamic trajectories over time rather than summary statistics covering broad analytic windows (eg, 6 or 12 months). In this analysis, we aimed to describe clinically actionable patterns of longitudinal PrEP persistence to inform the design of future PrEP persistence interventions.

## METHODS

### Study Population and Baseline Characteristics

The target population was adult MSM PrEP users (aged 18–65 years) in the United States. We identified approximately 17 000 adult male patients initiated in 2017 from the prescription fill database of a national chain pharmacy. Given the constrained computational capacity of our data systems, we employed an iterative stratified sampling method to select our final sample of 4000 individuals, which maximizes distributional representativeness compared to the source population. Details of the sampling method are provided in the Supplementary Content.

A tenofovir disoproxil fumarate and emtricitabine monotherapy (TFM) prescription was identified for PrEP initiation if it satisfied the following criteria: (1) the earliest prescription fill with ≥30-day supply in 2017 or with <30-day supply in 2017 that occurred before the earliest ≥30-day TFM in the same year, and (2) no prescription fills of antiretroviral medications existed between 1 January 2016 and the identified PrEP initiation date in 2017. The requirement of monotherapy excludes HIV treatments, which require other antiretroviral medications. The requirement of TFM with ≥30 days of supply was intended to prevent HIV postexposure prophylaxis prescription from being falsely identified as PrEP initiation. We assumed that if a patient's first TFM had <30-day supply and was followed by a TFM with ≥30 days of supply in that same year, the prescription was likely PrEP, as opposed to postexposure prophylaxis.

For each patient, we extracted the TFM prescription fill records that occurred between the PrEP initiation date and the earliest of the following 3 dates: (1) the end of a 2-year follow-up period since the PrEP initiation, (2) the earliest date of HIV antiretroviral therapy prescription fill records, and (3) the proxy HIV diagnosis dates, if collected from the patient.

We extracted individual-level demographic, financial, and pharmacy utilization information from a commercial pharmacy database. Community characteristics of patients at the aggregated level of the first 3 digits in the Zone Improvement Plan Code (ZIP-3) were extracted from the 2015–2019 US Census American Community Survey [8]. Characteristics (Supplementary Table 1) were selected based on data availability and observed associations with PrEP use from existing literature [9–12] . We were unable to identify MSM from the extracted male PrEP users. The impact of this limitation was likely minor because PrEP use is exceedingly rare among heterosexual adult males, with <1% of heterosexual active adults reporting PrEP use [13].

This analysis was approved by the Quorum institution review board, encompassing waivers for Health Insurance Portability and Accountability Act authorization and informed consent. The outputs of this analysis, shared with Emory University, were fully deidentified.

### Preprocessing of PrEP Prescription Data

The main challenge in inferring PrEP persistence patterns from prescription data is that it may not reflect the actual dosing of PrEP. Findings from a large online survey of MSM suggest that approximately 95% of PrEP users follow daily dosing [14]. Therefore, in the base analysis, we assumed each PrEP user followed a daily dosing schedule from the prescription refill date and would only obtain a new refill after exhausting their current supply.

Starting from each patient's PrEP initiation date, we computed the proportion of days covered by PrEP medication intake within biweekly intervals over 2 years under weekly moving strides. The biweekly intervals roughly correspond to the duration of PrEP cessation beyond which protection from HIV becomes poor [15]. We refer to this metric as biweekly proportions of moving coverage or PMC. The PMC time series was converted to the time series of PrEP seroprotection using the minimum effective PrEP adherence threshold (ie, 4 pills per week, or PMC of 57%) as a binary cutoff.

### Group-based Trajectory Modeling

Group-based trajectory modeling (GBTM) is a special case of finite mixture modeling, widely used to identify latent distinct clusters of individuals following similar trajectories [16]. GBTM assumes that observed trajectories arise from the realization of a finite mixture of prespecified distributions. In this analysis, the following probit model [17] was used to model the conditional distribution of the PrEP seroprotection, given group membership.


(1) $$P(y_{itj}=1)=\Phi \left(\beta_{0j}+\sum_{\;p=1}^{2}\beta_{\;pj}Time_{it}^{p}\right)$$

where $y_{itj}$ denotes whether a PrEP user *i* was considered sufficiently protected by PrEP over the next 2 weeks at week *t* since the initiation of PrEP, given that user *i* belongs to group *j*, *Φ* denotes the cumulative distribution function of a standard normal distribution, $Time_{it}^{p}$ denotes the number of weeks since the PrEP initiation date.

Data management and analysis were conducted using Python 3.10 (libraries: pandas, scipy, sklearn) and R 4.2 (packages: “lcmm”, “LCTMtools”).

### Evaluation of PrEP Trajectory Cluster

We defined the optimal cluster solution as a K-group model that achieved balance between model fitness and complexity, provided distinct cluster membership, and offered clinically actionable interpretations. Internal validity was evaluated using Nagin's diagnostic criteria, commonly used in the existing GBTM literature [18–23]. The qualitative interpretability of a cluster solution was assessed by examining the shapes of group-specific trajectories and characterizing them in the context of medication-seeking behaviors. To maintain effective cluster interpretability, we assessed only the cluster solutions for up to 6 trajectory groups.

### Association Analyses

We used unregularized and LASSO multinomial logistic models, in which we regressed the GBTM-predicted group membership on baseline characteristics. By shrinking the regression coefficients toward zero [24], LASSO regularization encourages parsimonious selection of relevant characteristics that potentially explain distinct trajectory group membership. The optimal magnitude of the regularization parameter *λ* was determined by 10-fold cross-validation of multinomial deviance (Supplementary Figure 1). To facilitate the interpretability of the regularized regression coefficients, we adopted a grouped LASSO penalty on all predictor variables.

## RESULTS

### Individual-level and Neighborhood-level Characteristics

Table 1 describes the distributions of the selected baseline characteristics among the 4000 sampled male PrEP users who initiated in 2017. On average, patients were 34.6 (standard deviation [SD] 10.5) years of age, with 15% aged 18–24 years. The average monthly PrEP copay amount during the 2-year follow-up time was $10.2 (SD 51.0). Approximately two-thirds did not incur any out-of-pocket costs for the PrEP prescriptions. Almost three-quarters of PrEP users had commercial insurance coverage and 18% had government insurance or assistance programs. The majority (88%) of patients filled PrEP prescriptions at traditional retail pharmacies. On average, 18% (SD 12%) and 25% (SD 15%) of the residents from the PrEP users’ neighborhood (ZIP-3 level) identified themselves as Black and Latinx/Hispanic, respectively. The average density of PrEP providers in the PrEP users’ neighborhood was 1.8 (SD 1.5) providers per 100,000 population. Consistent with the geographic distribution of the overall PrEP user population in the United States [25], the majority of patients were from the South (29.4%) and West (28.8%) regions.

**Table 1.** Patient-level and Neighborhood-level Characteristics

Characteristics	Study Sample (n = 4000)	
	Mean (SD)^a^ or N (%)^b^	
Demographics		
Age at PrEP initiation (y)^b^		
18–24	612	(15%)
25–29	969	(24%)
30–39	1307	(33%)
40–49	640	(16%)
50+	472	(12%)
Age at initiation (y)^a^	34.6	(10.5)
Average copay per month^b^		
$0	2586	(65%)
>$0	1414	(35%)
Average copay per month^a^	10.2	(51.0)
Pharmacy type^b^		
Specialty pharmacy	470	(12%)
Traditional retail pharmacy	3530	(88%)
Primary payer^b^		
Commercial	2922	(73%)
Government	732	(18%)
Cash/other	346	(9%)
PrEP use behaviors		
Duration under suboptimal seroprotection^b^		
<3 mo	1158	(29%)
3–6 mo	518	(13%)
6–9 mo	438	(11%)
9–12 mo	435	(11%)
12–15 mo	473	(12%)
15–18 mo	433	(11%)
18–21 mo	392	(10%)
21–24 mo	153	(4%)
Number of PrEP protective interval^b^		
1–3	2094	(52%)
4–6	1451	(36%)
7–9	395	(10%)
10+	60	(2%)
ZIP-3 level characteristics		
% Black residents^a^	17.9%	(12.1%)
% Latinx/Hispanic resident^a^	25.2%	(14.9%)
% Bachelor's degree or higher^a^	39.5%	(12.1%)
% Poverty^a^	13.4%	(3.5%)
% Uninsured^a^	10.5%	(5.5%)
PrEP provider density (per 100,0000 population)^a^	1.8	(1.5)
Geographic region^b^		
South	1177	(29.4%)
West	1152	(28.8%)
Midwest	869	(21.7%)
Northeast	794	(19.9%)
Other	8	(0.2%)

Abbreviations: PrEP, preexposure prophylaxis; SD, standard deviation.

^a^Mean and SD were computed for continuous variables.

^b^Sample size and proportion were obtained for each level in categorical variables.

For the 2-year follow-up period after PrEP initiation, the median duration under suboptimal PrEP seroprotection (ie, <57% of an interval covered by PrEP intake) was 35.0 (interquartile range 10–64) weeks. Approximately half of PrEP users had 4 or more separate intervals with optimal PrEP seroprotection during the follow-up period (Table 1), indicating either periodicity or imperfect regularity of PrEP use.

### PrEP Persistence Trajectories

Supplementary Figure 2 *A* and 2*B* display the trajectories of the predicted probabilities and observed proportion of optimal seroprotection by group membership for each evaluated K-group model. All K-group models (excluding the reference 1-group model) met all recommended model fitness criteria: average posterior probabilities of group membership (≥0.70), “Odds of Correct Classification” (≥5), and group sample size (each group presented ≥5% of the samples) (Supplementary Table 2). The scree plots of log-likelihood, Bayesian Information Criterion, and sample size-adjusted Bayesian Information Criterion show that benefits for improving model fitness (relative to the increased complexity of adding more groups) tapered down at k = 4–5 (Supplementary Figure 3). We opted to proceed with the 4-group model as the final solution because the resulting clusters elucidated distinct and easily interpretable PrEP use behaviors over time. We further examined how uncertainty in the assumption of daily PrEP use affected trajectory clusters in a sensitivity analysis, finding a negligible impact on cluster inference (Supplementary Table 3 and Supplementary Figures 4 and 5*B*).

**Figure 1.**
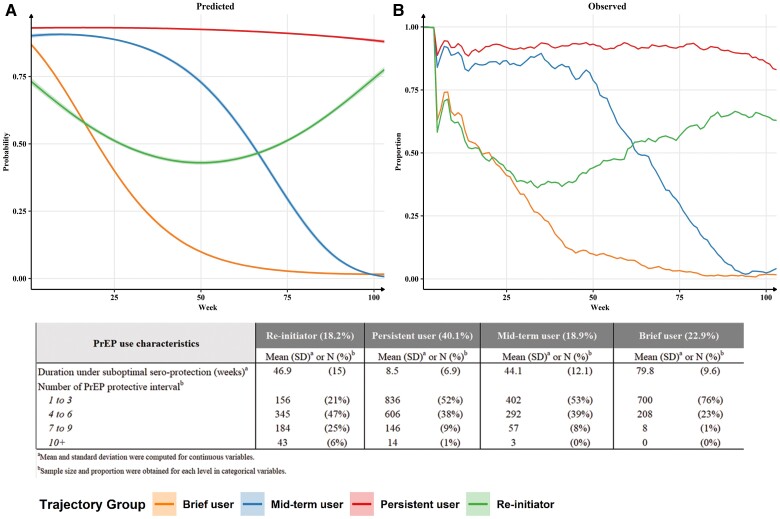
Predicted probabilities (Part A) and observed proportions (Part B) of optimal PrEP seroprotection and the corresponding PrEP use characteristics by group membership based on the fitted 4-group GBTM.

The following distinct PrEP persistence trajectory groups were identified in the 4-group model (Figure 1):

1. **Persistent user:** A high probability (>80%) of optimal PrEP seroprotection was consistently maintained throughout the 2-year follow-up period. The trajectory group represented 40% of the sample.
2. **Brief user:** The probability of optimal PrEP seroprotection declined rapidly after PrEP initiation. The trajectory group represented 23% of the sample.
3. **Reinitiator:** The probability of optimal PrEP seroprotection first dipped below 50% around 6 months after PrEP initiation but steadily returned to approximately 75% at the end of the follow-up period. The trajectory group represented 18% of the sample.
4. **Mid-term user:** The probability of optimal seroprotection was initially high but started to decline steadily in the second half of the first follow-up year. The trajectory group represented 19% of the sample.

On average, individuals assigned to the persistent user trajectory group spent only 8.5 (SD 6.9) weeks (∼2 months) under suboptimal PrEP seroprotection in the 2-year follow-up period (Figure 1). In contrast, individuals assigned to the brief user trajectory group spent 79.8 (SD 9.6) weeks (∼18.5 months) under suboptimal PrEP seroprotection, on average. There was no meaningful difference in the average duration under suboptimal protection between the reinitiator and mid-term user groups (46.9 weeks [SD 15.0] versus 44.1 weeks [SD 12.1]). Compared to the other trajectory groups, the reinitiator group had the greatest number of PrEP protective intervals because of more frequent PrEP reinitiation events.

### Correlates of PrEP use Trajectory Groups

**Figure 2.**
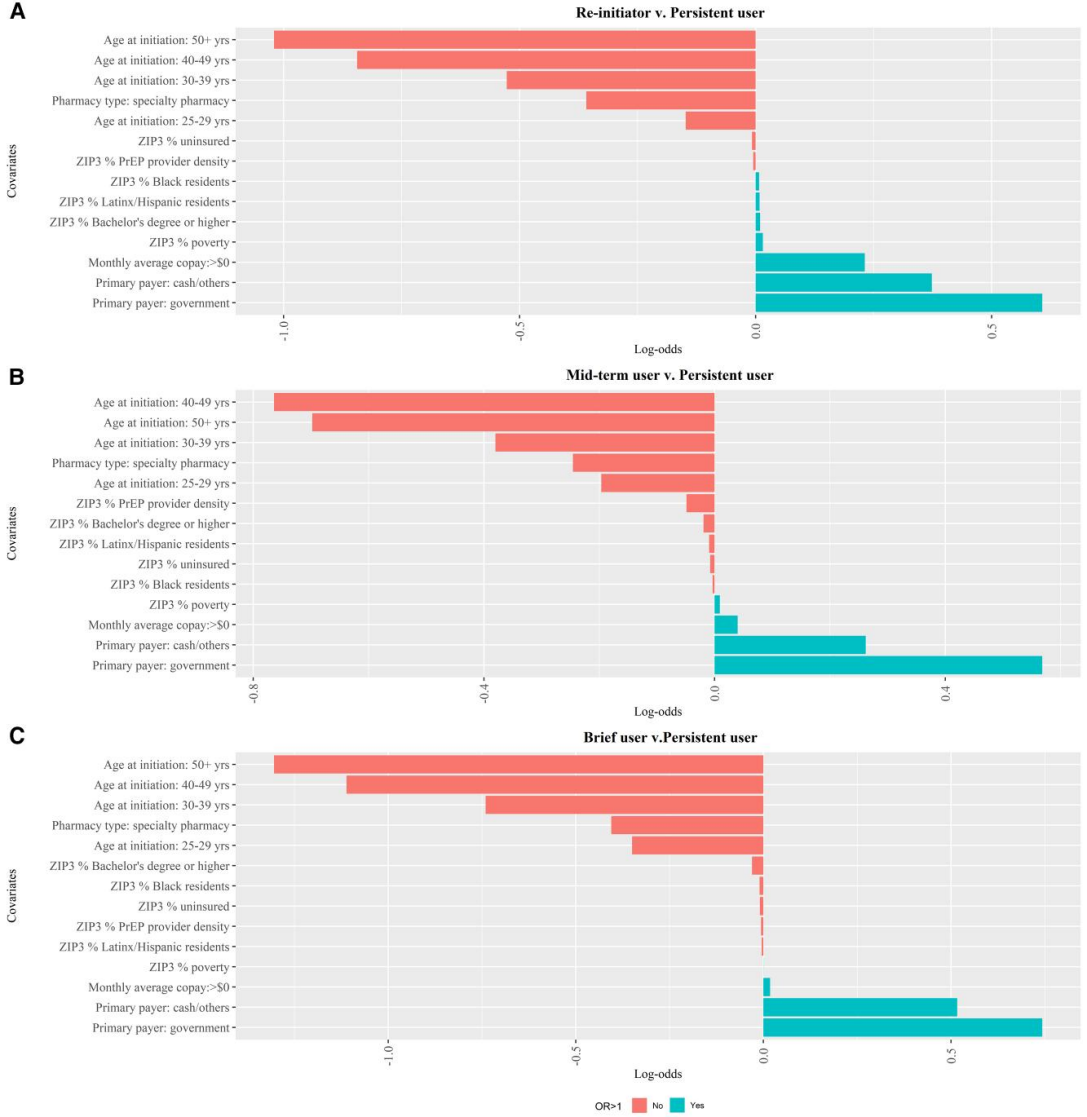
Penalized log-odds differences of demographic and ZIP-3 characteristics on the assigned trajectory group membership, LASSO multinomial logistic model.

Patients in the persistent user trajectory group were, on average, the oldest when initiating PrEP (36.8 [SD 10.5] years), had the highest proportion with commercial insurance as the primary payer (80%), and the highest proportion using specialty pharmacies (14%), compared with those in other trajectory groups (Table 2). Our multinomial logit model suggests that these characteristics were associated with the persistent PrEP use trajectory (Table 3). This is consistent with the LASSO model results, which show that age at initiation, primary payer and pharmacy type consistently had stronger adjusted associations than other covariates in all 3 binary regressions (Figure 2). Compared to reinitiators and mid-term users, patients in the brief user group, on average, were younger at PrEP initiation (32.3 [SD 10.1] years) and had a lower proportion with commercial insurance (66%). However, there was no substantial difference in the proportion using specialty pharmacy among patients in the brief user trajectory group (10%) compared to the reinitiator group (10%) and mid-term user group (12%) (Table 2).

**Table 2.** Descriptive Statistics of Characteristics by PrEP Persistence Trajectory Group Membership

Characteristics	Reinitiator		Persistent User		Mid-term User		Brief User	
	Mean (SD)^a^ or N (%)^b^		Mean (SD)^a^ or N (%)^b^		Mean (SD)^a^ or N (%)^b^		Mean (SD)^a^ or N (%)^b^	
Demographics								
Age at PrEP initiation (y)^b^								
18–24	130	(18%)	151	(9%)	122	(16%)	209	(23%)
25–29	205	(28%)	332	(21%)	185	(25%)	247	(27%)
30–39	229	(31%)	548	(34%)	255	(34%)	275	(30%)
40–49	99	(14%)	326	(20%)	103	(14%)	112	(12%)
50+	65	(9%)	245	(15%)	89	(12%)	73	(8%)
Age at PrEP initiation (y)^a^	33.0	(9.9)	36.8	(10.5)	34.3	(10.4)	32.3	(10.1)
Average copay per month^b^								
$0	440	(60%)	1043	(65%)	491	(65%)	612	(67%)
>$0	288	(40%)	559	(35%)	263	(35%)	304	(33%)
Pharmacy type^b^								
Traditional retail pharmacy	657	(90%)	1382	(86%)	667	(88%)	824	(90%)
Specialty pharmacy	71	(10%)	220	(14%)	87	(12%)	92	(10%)
Primary payer^b^								
Commercial	509	(70%)	1281	(80%)	532	(71%)	600	(66%)
Government	153	(21%)	203	(13%)	158	(21%)	218	(24%)
Cash/other	66	(9%)	118	(7%)	64	(8%)	98	(11%)
ZIP-3 level characteristics								
% Black residents^a^	18.3%	(11.9%)	17.8%	(11.9%)	17.9%	(12.5%)	17.7%	(12.1%)
% Latinx/Hispanic resident^a^	25.5%	(15.3%)	25.3%	(14.6%)	24.8%	(14.8%)	25.1%	(15.3%)
% Bachelor's degree or higher^a^	40.2%	(12.1%)	39.8%	(12.1%)	38.7%	(12.0%)	39.0%	(12.2%)
% Poverty^a^	13.5%	(3.5%)	13.3%	(3.4%)	13.4%	(3.4%)	13.3%	(3.6%)
% Uninsured^a^	10.2%	(5.4%)	10.6%	(5.5%)	10.5%	(5.3%)	10.3%	(5.4%)
PrEP provider density (per 100,0000 population)^a^	1.91	(1.56)	1.85	(1.55)	1.69	(1.51)	1.82	(1.55)

Abbreviations: PrEP, preexposure prophylaxis; SD, standard deviation.

^a^Mean and SD were computed for continuous variables.

^b^Sample size and proportion were obtained for each level in categorical variables.

**Table 3.** Associations of PrEP Persistence Trajectory Group Membership With Demographic and ZIP-3 Level Characteristics, Multivariate Multinomial Logistic Regression Model

Characteristics	Reinitiator vs. Persistent User				Mid-term User vs. Persistent User				Brief User vs. Persistent User			
	aOR	95% CI		*P*	aOR	95% CI		*P*	aOR	95% CI		*P*
Demographics												
Age at PrEP initiation (y)												
18–24	ref	…		…	ref	…	…	…	ref	…	…	…
25–29	0.728	.543	.977	.035	0.705	.522	.952	.023	0.550	.420	.720	<.001
30–39	0.494	.372	.656	<.001	0.595	.448	.789	<.001	0.375	.290	.485	<.001
40–49	0.358	.258	.498	<.001	0.399	.287	.555	<.001	0.252	.186	.342	<.001
50+	0.295	.205	.425	<.001	0.428	.303	.605	<.001	0.204	.145	.287	<.001
Average copay per month												
$0	ref	…		…	ref	…	…	…	ref	…	…	…
>$0	1.341	1.107	1.624	.003	1.033	.853	1.251	.737	1.020	.849	1.225	.836
Pharmacy type												
Traditional retail pharmacy	ref	…		…	ref	…	…	…	ref	…	…	…
Specialty pharmacy	0.667	.491	.907	.010	0.753	.565	1.004	.053	0.630	.474	.836	.001
Primary payer												
Commercial	ref	…		…	ref	…	…	…	ref	…	…	…
Government	1.934	1.517	2.465	<.001	1.831	1.442	2.326	<.001	2.193	1.752	2.745	<.001
Cash/other	1.589	1.140	2.214	.006	1.366	.980	1.903	0.065	1.830	1.356	2.469	<.001
ZIP-3 level characteristics												
% Black residents^a^	1.024	.970	1.082	.388	0.986	.935	1.039	.598	0.981	.932	1.032	.451
% Latinx/Hispanic residents^a^	1.028	.980	1.077	.256	0.982	.939	1.028	.440	0.994	.952	1.038	.780
% Bachelor's degree or higher^a^	1.028	.965	1.094	.397	0.988	.929	1.050	.691	0.948	.894	1.006	.078
% Poverty^a^	1.030	.848	1.251	.765	1.079	.894	1.303	.426	0.998	.832	1.197	.984
% Uninsured^a^	0.949	.823	1.094	.467	0.978	.853	1.122	.753	0.970	.849	1.108	.652
PrEP provider density (per 100,0000 population)	0.966	.882	1.059	.463	0.920	.839	1.007	.072	1.010	.926	1.101	.830

Abbreviations: aOR, adjusted odds ratio; CI, confidence interval; PrEP, preexposure prophylaxis; ZIP-3, first 3 digits in the Zone Improvement Plan Code.

^a^Effect per 5% increment.

## DISCUSSION

In this study, we mined longitudinal PrEP prescription fill data to address the knowledge gap regarding PrEP persistence patterns over extended intervals. Our study provides clinically relevant interpretations of PrEP persistence trajectory patterns linked to the time-varying risk of suboptimal PrEP seroprotection. Overall, we observed even declines (∼10%) of PrEP seroprotection at quarterly intervals after the first 3 months of PrEP use. This trend is consistent with the natural pinch points along the standard PrEP care routine, with patients needing to attend quarterly clinical visits and confirm PrEP indication to continue their prescription. This finding may highlight the importance of considering the inherent barriers associated with regular PrEP clinical visits (eg, concerns about stigma, lack of nearby facilities, time consuming in-person appointments and laboratory procedures [26]).

Furthermore, we identified 4 distinct groups of PrEP persistence trajectories (ie, persistent users, brief users, reinitiators, and mid-term users) that may be instrumental in characterizing heterogeneity in PrEP use behaviors over time. A number of existing trajectory clustering studies (Pasipanodya et al [27], Sagaon-Teyssier et al [21], and Pyra et al [28]) similarly identify persistent and brief use trajectory groups. This suggests a strong presence of these 2 contrasting behavioral patterns in PrEP use. Comparatively, persistence estimated from randomized controlled trial settings [21, 27] was marginally greater than that observed in our study. This is expected because real-world medication adherence tends to be lower than that in study settings [29–31]. Trial participants universally receive incentives to attend study visits and receive free access to PrEP, which mitigates well-documented financial and logistical barriers to PrEP care [32].

The prevalence of a brief user trajectory was marginally lower than that identified with the “short PrEP use” trajectory (30%) in Pyra et al [27]. It is worth noting that the patients in Pyra et al [28] were followed for only 12 months. PrEP persistence for the mid-term user trajectory group and the reinitiator trajectory group did not start declining and recovering, respectively, toward the end of the first year of follow-up. Therefore, the slightly larger group membership proportion for the “short PrEP use” trajectory could be due to the inclusion of individuals whose PrEP persistence would have recovered if the follow-up time extended. Furthermore, if we assume our study population follows a similar racial distribution as the broader PrEP user population in the United States (ie, 11% are Black [25]), the lower prevalence of the sustained low persistence trajectory in our study could potentially be due to the likely lower proportion of black PrEP users, who are at elevated risk for low PrEP persistence because of systemic barriers to PrEP care [33, 34–37], in our study sample compared to theirs (ie, >40%).

Our study further illuminates the heterogeneity within PrEP persistence patterns that exists between these 2 prominent but contrasting modes of PrEP use behaviors. We found that PrEP users may be characterized either by their tendency to reinitiate PrEP following cessations (ie, reinitiator trajectory group) or to engage in a longer period of initial PrEP use followed by cessation (ie, mid-term user trajectory group). The distinction between these 2 trajectory groups suggests the potential for optimizing the timing of PrEP persistence interventions to maximize their benefits for different sets of PrEP users.

Assuming that PrEP users are constantly exposed to potential HIV risks, approximately 20% the mid-term user trajectory group members could benefit from PrEP persistence interventions focused on follow-ups at 12–15 months after PrEP initiation. On the other hand, around 40% of the individuals (ie, brief user trajectory group and the reinitiator trajectory group) could benefit from similar interventions targeting the initial 6 months after initiation. The utility of these key intervention time points may need to be further validated against the patterns of time-varying exposure to potential HIV risk. This is because patients’ decisions to continue PrEP use may also be influenced by their sexual activities and risk behaviors, which determines their need for PrEP, in addition to perceived barriers to obtaining refills. Depending on which factor primarily drives the persistence gaps observed in this study, the implication for the timing or type of intervention needed may differ.

In this study, we found that use of specialty pharmacy, older age at PrEP initiation, and having commercial insurance as the primary payer of PrEP were associated with a persistent PrEP use trajectory. This is consistent with findings from a prior study that assessed the correlates of annual PrEP persistence among users within the same national pharmacy chain [38]. The positive association between attending specialty pharmacy and persistent use trajectory could be due to the comprehensive support services provided by the specialty pharmacy for patients requiring complex medication management. Compared to traditional retail pharmacies, specialty pharmacy staff generally receives further training in financial assistance navigation, medication synchronization, and drug adherence support. In addition, patients at a specialty pharmacy often receive longer and more personalized consultation compared to those at traditional pharmacies. Other studies have similarly documented an association between improved medication adherence and the use of specialty pharmacies [39, 40]. In addition, barriers to PrEP use among young MSM have been well documented (eg, financial vulnerability, low perception of HIV risk, challenges to navigate complex health systems) [38, 41, 42]. Lacking health insurance coverage has also been associated with low PrEP use in other studies [6, 11, 28, 34, 43]. Given these findings, it may be beneficial to prioritize targeting and tailoring PrEP persistence interventions for patients aged <25 years who lack access to commercial insurance and specialty pharmacies (ie, most at risk of nonpersistence).

### Limitations

First, we used prescription fill records to approximate actual PrEP intake history, which could not be easily collected by pharmacies. This may have led to overestimation of PrEP persistence, as we assumed strict adherence to daily intake given a refill. Nevertheless, such an overestimation would likely be limited to a single refill period because patients who refilled prescriptions without consuming them were unlikely to return for the next refill. Furthermore, our sensitivity analysis suggests the robustness of the identified trajectories against the uncertainty around PrEP intake habits.

Second, we lack the information regarding the patients’ true PrEP indication statuses (eg, sexual activities, risk behaviors) over time. Consequently, we could not further derive the typology of HIV risk trajectories, which limit the applicability of the identified PrEP persistence trajectories for developing interventions that target the dynamic nature of HIV risk. Another limitation resulting from data restriction is that we could not empirically validate whether the observed suboptimal PrEP seroprotection was truly PrEP use cessation. This could lead to misclassification bias for individuals who switched to a different pharmacy chain during their follow-up period. This was because they would have been considered to have suboptimal seroprotection by our data mechanism, although they may adhere to PrEP medication prescribed from other pharmacies. Nevertheless, a previous study from the same pharmacy chain found that >80% of the PrEP users consistently accessed the collaborating pharmacy chain ever after their final refills. Furthermore, no substantial differences in annual PrEP persistence between the overall study sample and this subset of patients. These findings suggest that patients were likely not switching pharmacies at a high rate to substantially alter our findings.

Another limitation to our study is that our definition of PrEP regimen does not exclude TFM used for chronic hepatitis B management. This could lead to misclassification in rare instances because such prescription use is not clinically recommended. In addition, our study population consisted of patients obtaining PrEP medication through commercial pharmacies, the majority of whom have private insurance coverage. They may have different characteristics compared to patients who obtain PrEP elsewhere or those from socioeconomically marginalized populations. This could potentially limit the transportability of the observed PrEP persistence patterns and associations to other PrEP user populations. Our payer data also lacks full granularity with regard to the source of payment for PrEP. This hinders us from evaluating the use of copay assistance program (independently from insurance coverage or as a primary payer type) in relation to PrEP persistence patterns. Furthermore, our limited access to pharmacies’ business and operational data (eg, drug dispensing method, locational data) does not allow us to further validate the primary mechanisms by which use of specialty pharmacy is associated with increased PrEP persistence. We also could not assess PrEP use trajectories by geographic regions due to limited data use agreement. Last, the observed PrEP persistence patterns may not be transportable to long-acting injectable PrEP patients, who are recommended to receive injections every 2 months, as opposed to monthly refills.

## CONCLUSION

By fitting GBTM to PrEP seroprotection coverage derived from pharmacy refill data, our study contributes to the understanding of the heterogeneous patterns of PrEP persistence among male users. We identified 4 qualitatively distinct PrEP persistence trajectory groups: (1) persistent user, (2) brief user, (3) mid-term user, and (4) reinitiator. Notably, the subgroup characterized by persistent PrEP use emerged as the largest (∼40% of the PrEP users), whereas the remaining 3 subgroups shared comparable group sizes. Younger age at PrEP initiation and noncommercial primary health insurance exhibited the strongest associations with various patterns of PrEP nonpersistence. Furthermore, our study highlights the significance of specialty pharmacy coverage in enhancing the persistence of PrEP. This underscores the need for consistent support, encompassing both financial and behavioral aspects, throughout an individual's PrEP journey, especially during the critical time points of PrEP cessation identified in this study.

## Supplementary Material

ofae584_Supplementary_Data

## Supplementary Data

Supplementary materials are available at *Open Forum Infectious Diseases* online. Consisting of data provided by the authors to benefit the reader, the posted materials are not copyedited and are the sole responsibility of the authors, so questions or comments should be addressed to the corresponding author.

## Notes

***Financial support*.** During the period of working on this analysis, the doctoral stipend of the first author was funded by a research project grant from the National Institute of Health (R01MH114692).
