# Supplementary material for: Understanding Typology of Preexposure Prophylaxis (PrEP) Persistence Trajectories Among Male PrEP Users in the United States
Source: Open Forum Infect Dis. 2024 Oct 11;11(11):ofae584. doi: 10.1093/ofid/ofae584 (PMC11574613; doi:10.1093/ofid/ofae584)
Supplement: ofae584_Supplementary_Data [file ofae584_supplementary_data.docx]

## Supplemental Contents

### Section 1: Iterative stratified sampling

We iteratively implemented stratified sampling for 100 times based on the following covariates: 1) age at PrEP initiation; 2) average monthly copay; 3) primary payer type; 4) duration under sub-optimal PrEP sero-protection, derived from the binary PrEP sero-protection data matrix. Age at PrEP initiation, average monthly copay and primary payer type were selected because of their observed association with PrEP persistence in our previous study[1]. Duration under suboptimal PrEP sero-protection was calculated to summarize the overall PrEP persistence in the two-year follow-up period.

The 100 sampled datasets were then ranked based on how closely their bivariate distributions matched those of the source dataset. Root-mean square error (RMSE) was used to assess an average distance in spearmans’ correlation coefficients among covariate pairs where both are continuous variables. For covariate pairs where at least one variable is categorical, RMSE on the cross-tabulation frequencies were selected as a distance measure, which were then averaged among all the categorical covariate pairs. (When paired with a categorical variable, the continuous variable was divided into tertiles to enable the calculation of cross-tabulation frequencies.) The sample dataset with the best rank (weighted between continuous and categorical distance ranking) was selected as the study sample, or the working dataset for the subsequent trajectory clustering analysis.

### Section 2: Sensitivity analyses

We evaluated the impact of non-daily dosing schedules on the mined typology from the group-based trajectory model (GBTM). First, we would like to define new terms:

1. **PrEP assessment interval:** the time interval bounded by two consecutive PrEP prescription fill dates, or the end date of the two-year follow-up period.
2. **Adherent PrEP consumption period:** the duration between the start date of a PrEP assessment interval and the earlier of the following two dates: 1) the end date of the PrEP assessment interval or 2) the end date of a 7-day extension period after the end of the prescription’s days supply. Since most oral PrEP prescriptions are filled with a 30-day supply, the 7-day relaxation corresponds to a dosing frequency of approximately 6 doses per week. This fits the working definition of daily dosing schedule referenced in a nationwide survey study measuring PrEP dosing frequencies [2]. The adherent PrEP consumption period effectively approximated the duration a PrEP prescription fill would last if a PrEP user mostly maintain daily dosing frequency.

We assigned 10% of PrEP users to follow a nondaily dosing regimen. Non-daily PrEP users were randomly selected from the subset of PrEP users who had at least one PrEP assessment intervals in which the interval lengths were greater than the length of the adherent PrEP consumption periods. To preserve analysis parsimony, we assumed that all PrEP users completed their prescribed supply before the next PrEP assessment interval. The aforementioned selection process gave us two subpopulations of study subjects:

1. **Daily PrEP users:** all of their PrEP assessment intervals were assumed to strictly follow daily dosing regimen, as in the base analysis assumption.
2. **Non-daily PrEP users**: they were assumed to strictly follow the daily dosing regimen for the PrEP assessment intervals in which the interval lengths were within the adherent PrEP consumption periods. However, they were assumed to use a non-daily PrEP regimen for the PrEP assessment intervals in which the interval lengths were greater than those of the adherent PrEP consumption periods. A nationwide survey study on PrEP dosing frequency suggests that a large proportion of non-daily PrEP users followed an event-based PrEP dosing schedule (primarily on-demand 2-1-1 dosing regimen) or a regular schedule (e.g., every other day, on “T” [Tuesday, Thursday] and “S” [Saturday, Sunday]) [2]. Assuming MSM have roughly 1 sex per week as an average adult in the US [3], both event-based and regular non-daily dosing schedules are equivalent to following a PrEP dosing regimen of 4 doses/week. For the PrEP assessment intervals eligible for non-daily PrEP regimen, the PrEP intake dates were randomly selected between the interval start date and the earlier of the two following dates: interval end date or the date a prescription supply was expected to run out according to the 4 doses/week dosing schedule. The total number of selected PrEP intake dates in a PrEP assessment interval should equal the prescription days’ supply of that interval.

A modified proportion of moving coverage (PMC) and PrEP sero-protection time series matrices were created, following the selections of PrEP intake dates per the non-daily dosing schedule. The binary PrEP sero-protection time series matrix was analyzed using the same GBTM procedures as in the base analysis. Given the limited computational availability, we only implemented one iteration and compared the resulting iteration-specific cluster structures (i.e., the number of discovered groups, the proportion of group membership, and group-specific predicted trajectories) with that obtained in the base analysis.

All k-group models from the sensitivity analysis scenario meets the recommended model fitness criteria (Supplementary Table 3). The scree plots of BIC and sample-size-adjusted BIC also show similar decay pattern as K increases (Supplementary Figure 4), which indicates that the optimal K values for cluster partition remains around 4~5. When comparing the group-specific trajectories of optimal sero-protection probabilities at K=4~5 between the base and sensitivity analysis scenarios, we found minor variations in the probabilities in the early months of the follow-up period among trajectory groups characterized by consistently low sero-protection probabilities. For instance, when examining K=4, the Group 4 trajectory in the sensitivity analysis scenario (Supplementary Figure 5a) began with lower probability, and subsequently had a more gradual downward slope in comparison to its counterpart trajectory from the base analysis (Group 4 trajectory in Supplementary Figure 2a). This outcome was to be expected, given that individuals belonging to the sustained low sero-protection group typically displayed fewer and sporadic PrEP refills, which left more room for the introduction of variability to the non-daily PrEP intake dates through random assignment.

Nevertheless, the fundamental shapes of the optimal PrEP sero-protection probability trajectories remain consistent across both analysis scenarios (Supplementary Figures 2 & 5). This finding implies that the overall cluster structure of trajectories identified in the base analysis may be robust against the uncertainty around the daily PrEP dosing assumption.

### Section 3: Supplemental tables and figures

**Supplementary Table 1**. Individual-level and neighborhood-level characteristics selected for the association analysis with PrEP use trajectory patterns.

| Variable Name | Definition/Note | Variable Type | Level (if applicable) |
| --- | --- | --- | --- |
| **Individual-level characteristics** | | | |
| Age at PrEP initiation (yr) |  | Categorical | 1. 18 to 24 years 2. 25 to 29 years 3. 30 to 39 years 4. 40 to 49 years 5. 50+ years |
| Primary payer type | Source of payment used most frequently in the 2-year tracking period. If two sources of payments were received, the commercial or government insurance coverage will be prioritized over other copay assistance. | Categorical | 1. Commercial 2. Government (Medicaid, Medicare, Tricare/Veteran administration, health insurance exchange, 340B drug pricing program) 3. Cash/Other (including copay assistance) |
| Average copay per month (USD) | Average out-of-pocket monthly payment for medication over the duration PrEP medication was filled, excluding other cost categories such as lab tests and clinical visits | Categorical | 1. $0 2. > $0 |
| Pharmacy type |  | Categorical | 1. Specialty pharmacy 2. Traditional retail pharmacy |
| **ZIP-3-level characteristics** | | | |
| Concentrations of black populations |  | Continuous |  |
| Concentration of Latinx/Hispanic population |  | Continuous |  |
| Concentration of residents with Bachelor’s degree or higher |  | Continuous |  |
| Concentrations of residents under poverty threshold |  | Continuous |  |
| Concentrations of residents uninsured |  | Continuous |  |
| Density of PrEP provider[4] | Unit: per 100,000 people | Continuous |  |

**Supplementary Figure 1.** The 10-fold cross-validation multinomial deviance curve, by the log-transformed Lasso-regularization parameter ($\lambda$)


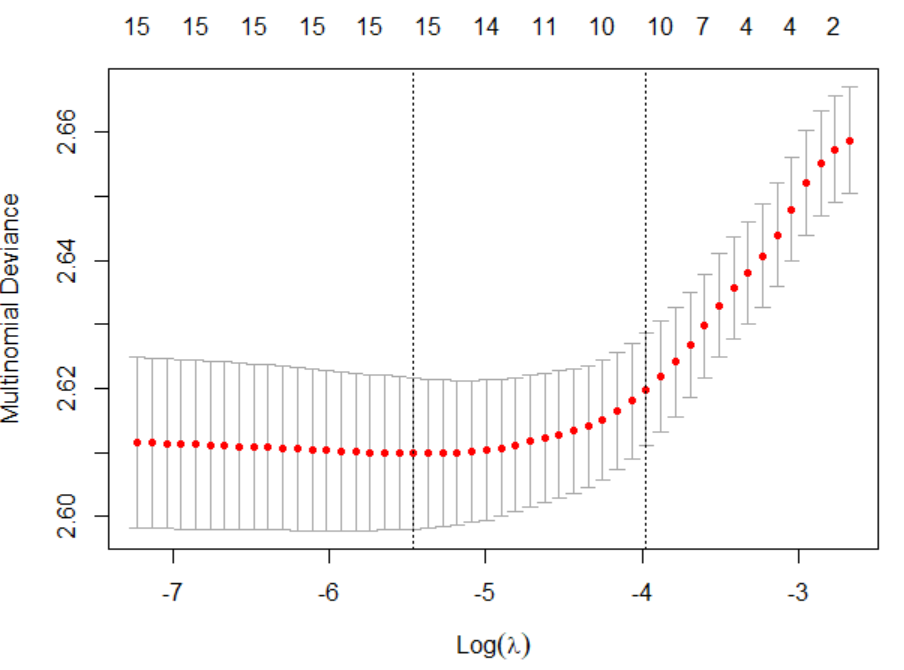


**Note:** the left vertical dotted line indicates the λ value that resulted in minimum mean deviance (i.e., optimal value of $\lambda$) and the λ value in the most regularized model from which the cross-validated deviance is within one standard error of the minimum.

**Supplementary Figure 2a.** Predicted probabilities of optimal PrEP sero-protection by assigned trajectory group membership (per the fitted group-specific probit regressions in GBTM)


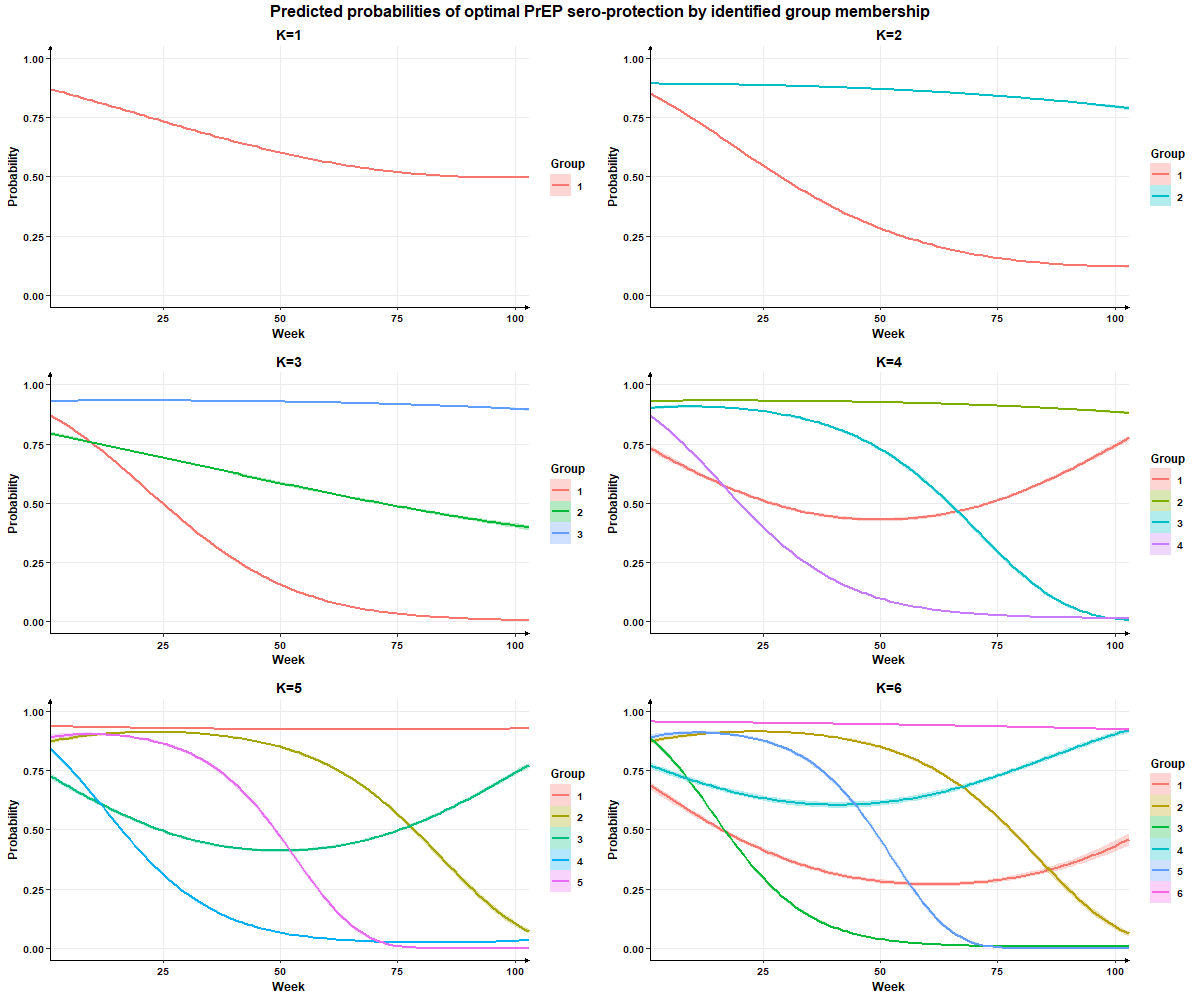


**Supplementary Figure 2b.** Observed proportion of PrEP users with optimal PrEP sero-protection by the trajectory group membership assigned by GBTM.


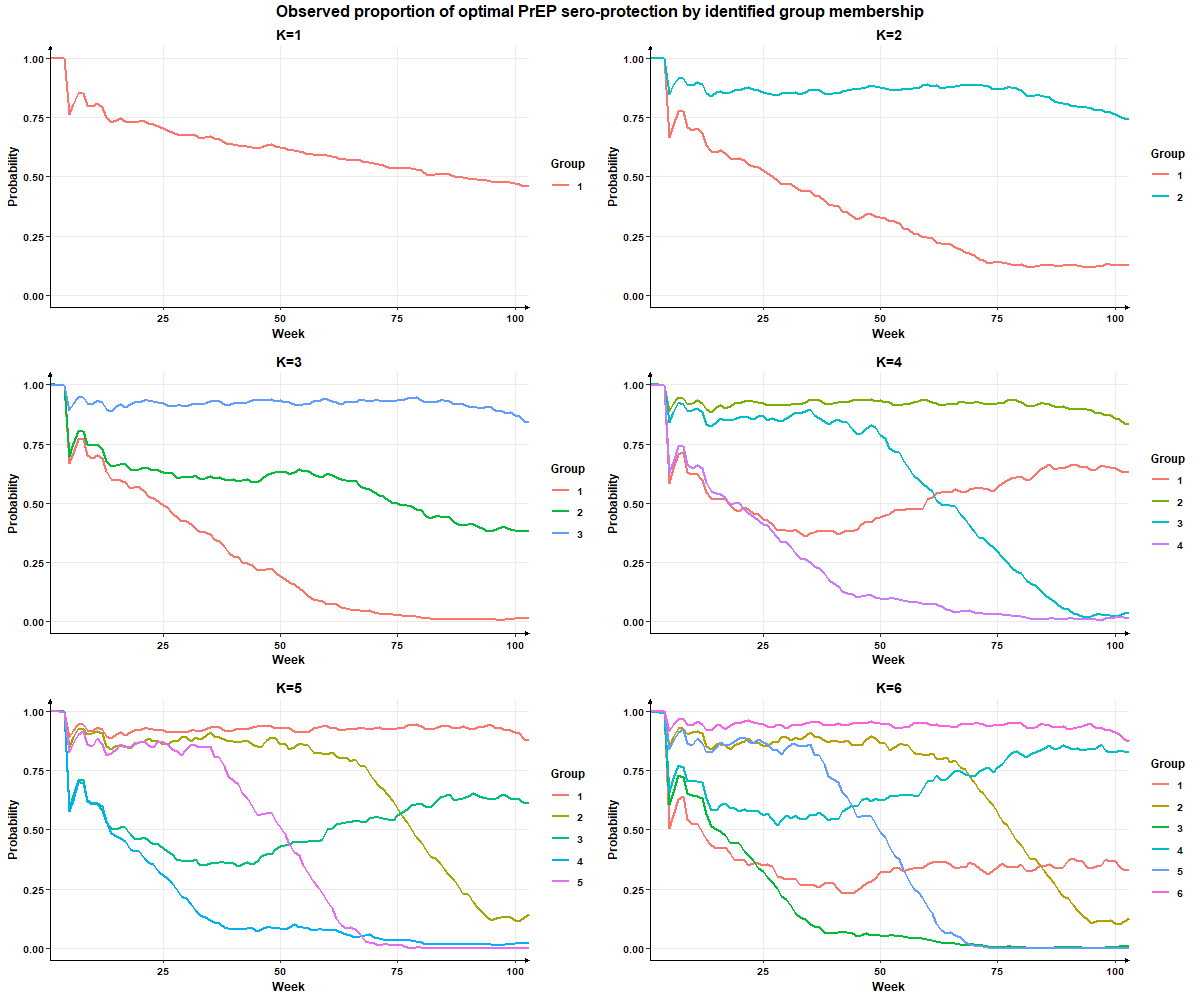


**Supplementary Table 2.** Goodness of fit indicators of GBTM models by various K values

| **K** | **Trajectory group membership** | | | | | | | | | | | | | | | | | |
| --- | --- | --- | --- | --- | --- | --- | --- | --- | --- | --- | --- | --- | --- | --- | --- | --- | --- | --- |
|  | **Group 1** | | | **Group 2** | | | **Group 3** | | | **Group 4** | | | **Group 5** | | | **Group 6** | | |
|  | *%^a^* | *APP^b^* | *OCC^c^* | *%^a^* | *APP^b^* | *OCC^c^* | *%^a^* | *APP^b^* | *OCC^c^* | *%^a^* | *APP^b^* | *OCC^c^* | *%^a^* | *APP^b^* | *OCC^c^* | *%^a^* | *APP^b^* | *OCC^c^* |
| 1 | 100.0% | 100.0% | - | - | - | - | - | - | - | - | - | - | - | - | - | - | - | - |
| 2 | 45.9% | 99.5% | 217.8 | 54.1% | 99.6% | 209.1 | - | - | - | - | - | - | - | - | - | - | - | - |
| 3 | 28.1% | 99.3% | 387.4 | 32.9% | 98.6% | 140.6 | 39.0% | 99.3% | 217.2 | - | - | - | - | - | - | - | - | - |
| 4 | 18.2% | 98.8% | 360.5 | 40.1% | 99.4% | 249.2 | 18.9% | 98.4% | 260.7 | 22.9% | 99.2% | 419.7 | - | - | - | - | - | - |
| 5 | 37.2% | 99.5% | 345.8 | 14.2% | 97.9% | 277.7 | 17.7% | 98.6% | 323.5 | 18.3% | 99.2% | 531.0 | 12.7% | 99.0% | 650.5 | - | - | - |
| 6 | 12.2% | 97.6% | 297.3 | 14.0% | 97.5% | 237.2 | 15.3% | 98.8% | 457.2 | 13.6% | 97.2% | 216.8 | 12.2% | 98.5% | 484.5 | 32.8% | 99.0% | 211.7 |
| ^a^ Proportion of persons assigned to a specific group membership | | | | | | | | | | | | | | | | | | |
| ^b^ Average posterior probability of group membership | | | | | | | | | | | | | | | | | | |
| ^c^ Odds of correct classification | | | | | | | | | | | | | | | | | | |

**Supplementary Figure 3.** Log-likelihood, Bayesian information criterion (BIC) and sample-size-adjusted BIC, by K values


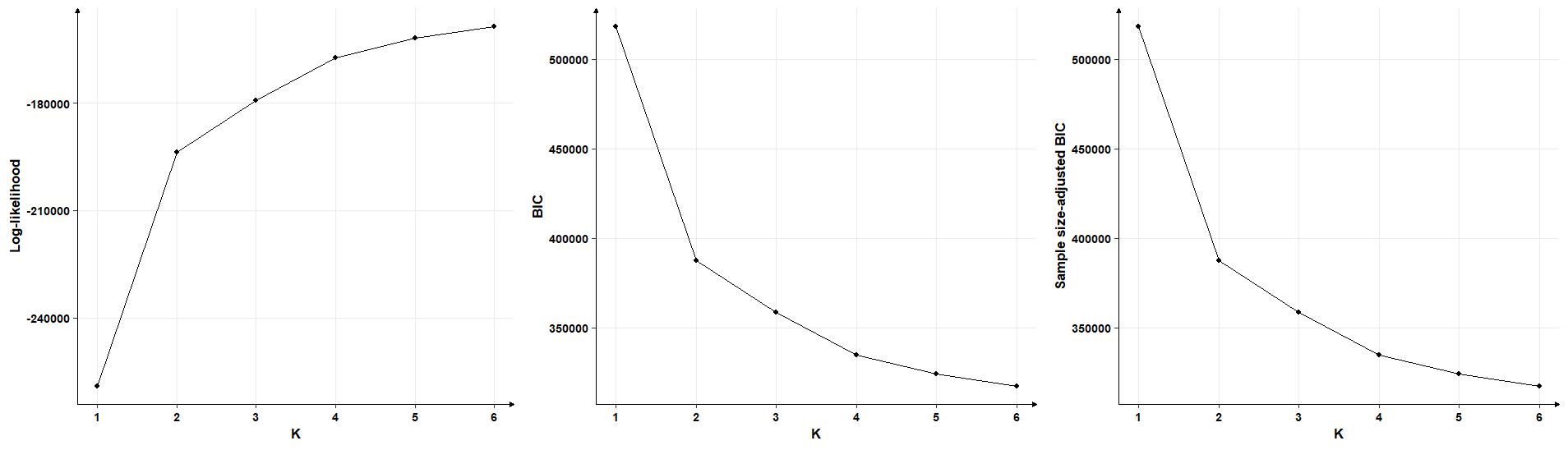


**Supplementary Table 3.** Sensitivity analysis: Goodness of fit indicators of GBTM models by various K values.

| **K** | **Trajectory group membership** | | | | | | | | | | | | | | | | | |
| --- | --- | --- | --- | --- | --- | --- | --- | --- | --- | --- | --- | --- | --- | --- | --- | --- | --- | --- |
|  | **Group 1** | | | **Group 2** | | | **Group 3** | | | **Group 4** | | | **Group 5** | | | **Group 6** | | |
|  | *%^a^* | *APP^b^* | *OCC^c^* | *%^a^* | *APP^b^* | *OCC^c^* | *%^a^* | *APP^b^* | *OCC^c^* | *%^a^* | *APP^b^* | *OCC^c^* | *%^a^* | *APP^b^* | *OCC^c^* | *%^a^* | *APP^b^* | *OCC^c^* |
| 1 | 100.0% | 100.0% | - | - | - | - | - | - | - | - | - | - | - | - | - | - | - | - |
| 2 | 48.8% | 99.5% | 341.2 | 51.2% | 99.7% | 330.9 | - | - | - | - | - | - | - | - | - | - | - | - |
| 3 | 43.7% | 99.3% | 363.4 | 33.5% | 99.3% | 278.5 | 22.8% | 98.8% | 286.8 | - | - | - | - | - | - | - | - | - |
| 4 | 18.5% | 98.8% | 278.0 | 37.4% | 99.4% | 291.3 | 16.5% | 98.2% | 276.1 | 27.8% | 99.2% | 315.8 | - | - | - | - | - | - |
| 5 | 13.5% | 99.5% | 442.9 | 14.8% | 98.1% | 291.6 | 35.0% | 99.3% | 272.5 | 14.8% | 98.4% | 354.9 | 22.0% | 99.0% | 368.2 | - | - | - |
| 6 | 11.1% | 97.6% | 429.9 | 33.2% | 99.3% | 307.4 | 13.7% | 97.8% | 283.9 | 14.0% | 98.1% | 311.1 | 10.0% | 98.2% | 484.3 | 18.1% | 98.6% | 329.5 |
| ^a^ Proportion of persons assigned to a specific group membership | | | | | | | | | | | | | | | | | | |
| ^b^ Average posterior probability of group membership | | | | | | | | | | | | | | | | | | |
| ^c^ Odds of correct classification | | | | | | | | | | | | | | | | | | |

**Supplementary Figure 4.** Sensitivity analysis: Log-likelihood, Bayesian information criterion (BIC) and sample-size-adjusted BIC, by increasing K values.


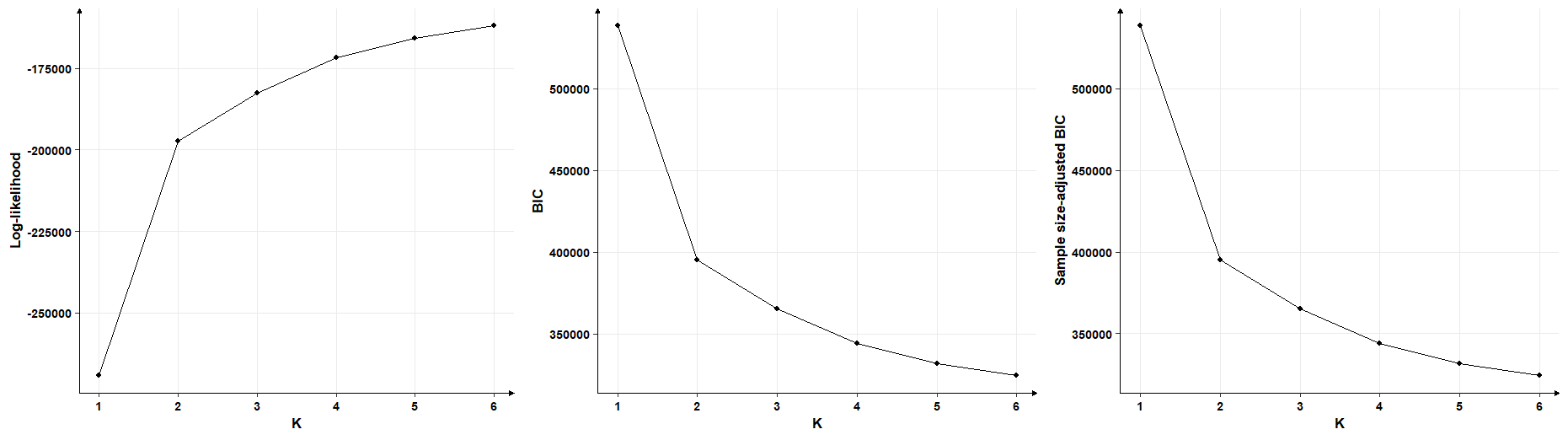


**Supplementary Figure 5a.** Sensitivity analysis: Predicted probabilities of optimal PrEP sero-protection by assigned trajectory group membership (per the fitted group-specific probit regressions in GBTM).


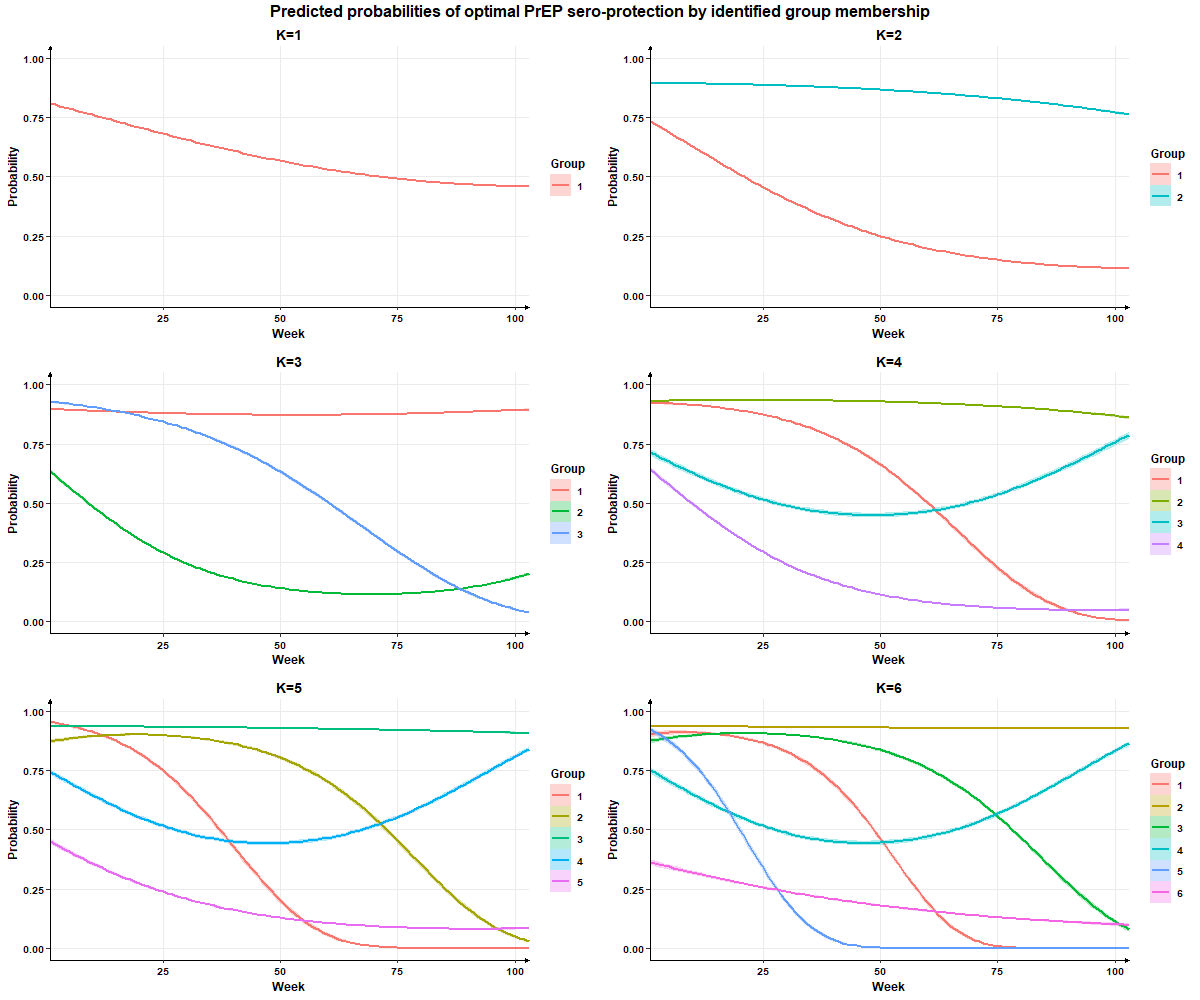


**Supplementary Figure 5b.** Sensitivity analysis: Observed proportions of optimal PrEP sero-protection by trajectory group membership assigned by GBTM.


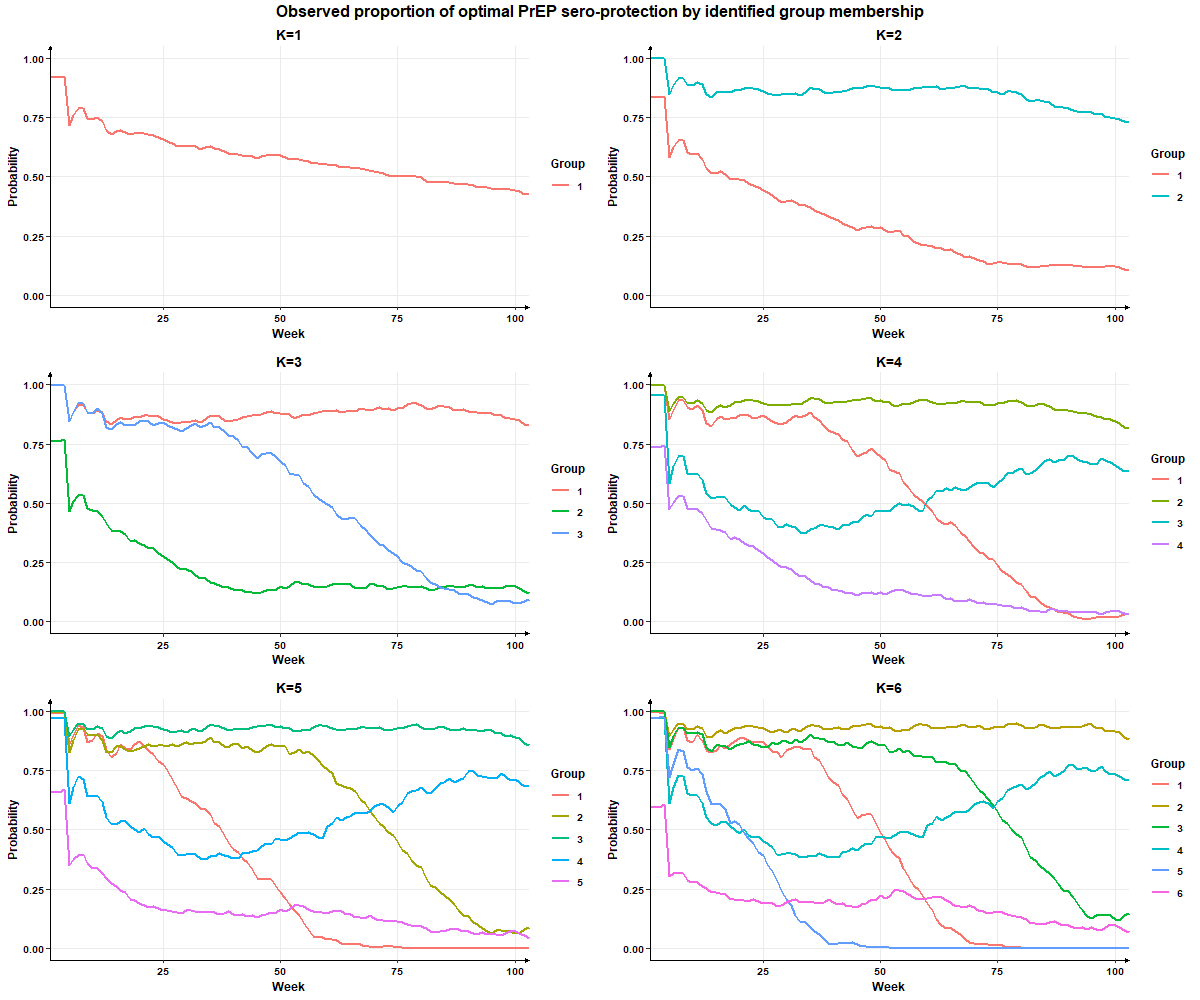


### Section 4: Reference

1. Coy, K.C., et al., *Persistence on HIV preexposure prophylaxis medication over a 2-year period among a national sample of 7148 PrEP users, United States, 2015 to 2017.* Journal of the International AIDS Society, 2019. **22**(2): p. e25252-e25252.

2. Sewell, W.C., et al., *Nondaily Use of HIV Preexposure Prophylaxis in a Large Online Survey of Primarily Men Who Have Sex With Men in the United States.* Journal of acquired immune deficiency syndromes (1999), 2020. **84**(2): p. 182-188.

3. Twenge, J.M., R.A. Sherman, and B.E. Wells, *Declines in Sexual Frequency among American Adults, 1989-2014.* Arch Sex Behav, 2017. **46**(8): p. 2389-2401.

4. Siegler, A.J., et al., *Developing a Web-Based Geolocated Directory of HIV Pre-Exposure Prophylaxis-Providing Clinics: The PrEP Locator Protocol and Operating Procedures.* JMIR Public Health Surveill, 2017. **3**(3): p. e58.
